# An In Vitro Comparison of the Antibacterial Efficacy of Triphala with Different Concentrations of Sodium Hypochlorite

**Published:** 2014-10-07

**Authors:** Sahar Shakouie, Mahsa Eskandarinezhad, Negin Gasemi, Amin Salem Milani, Mohammad Samiei, Sara Golizadeh

**Affiliations:** a *Department of Endodontics, Dental School, Tabriz University of Medical Sciences, Tabriz ,Iran;*; b *Private Practice, Tehran, Iran*

**Keywords:** Antibacterial Agents, *Enterococcus Faecalis*, Root Canal Treatment, Sodium Hypochlorite, Triphala, Zone of Inhibition

## Abstract

**Introduction:** The antimicrobial efficacy of root canal irrigant plays an important role in increasing the success of root canal treatment (RCT). The aim of the present experimental study was to compare the antimicrobial activity of Triphala (a plant-derived solution) with 0.5, 1, 2.5 and 5% concentrations of sodium hypochlorite (NaOCl), against *Enterococcus faecalis *(*E. faecalis*). **Methods and Materials:** Two hundred plates of cultured *E. faecalis*, were divided into 5 experimental groups (*n*=38) and two positive and negative control groups. The antimicrobial activity of the test solutions was determined by measuring the zone of inhibition in the culture media. The mean diameter of inhibited zones between the study groups was compared using the Kruskal-Wallis test and the Mann-Whitney U test was used for the two-by-two comparison of the groups with the level of significance set at 0.05. **Results: **The Kruskal-Wallis test showed significant differences between the study groups (*P*<0.05). According to the Mann-Whitney U test the mean diameter of inhibition zones in Triphala group was significantly higher compared to 0.5 and 1% NaOCl (*P*<0.05). **Conclusion: **In this study, Triphala exhibited better antimicrobial activity against *E. faecalis* compared to 0.5 and 1% NaOCl (*P*<0.05).

## Introduction

The principal aim of endodontic treatment is to debride and disinfect the root canal system. Mechanical instrumentation does not achieve this aim alone. Therefore, chemical disinfection by means of several irrigation solutions is recommended [1-4]. An ideal irrigant destroys bacteria, dissolves necrotic debris, lubricates the root canal and removes the smear layer without irritating healthy tissues [1, 5]. At present, NaOCl is the most common root canal irrigant. It is a strong proteolytic substance and provides sufficient antimicrobial effect [5-9]. However, adverse effects of NaOCl have been reported including unpleasant odor and taste, toxicity, possible paresthesia of the mandibular nerve, allergy and an increase in coronal microleakage of adhesive restorations [7-9].

*Enterococcus faecalis *(*E. faecalis*) is one of the most commonly isolated microorganisms from the failed endodontic treatments. Because of its adhesion to dentin and penetration into the dentinal tubules and resistance to the antimicrobial effects of calcium hydroxide (CH), elimination of this microorganism is very difficult, if not impossible [10].

Triphala [three (tri) fruits (phala)] is a plant-derived composition developed in India; the powder is a combination of three dried plants naming *Terminaliabellerica, Terminaliachebula* and *Emblicaafficinalia* with tanic acid being its principal constituent [11-13]. It has been used in Indian traditional medicine for treatment of headaches, constipation and hepatic disorders [12-16]. Initial studies have shown bacteriostatic or bactericidal effect of tanic acid on gram-positive and gram-negative pathogens [16]. Compared to commonly used root canal irrigants, it is safe and is composed of compounds with proper physiologic effects in addition to its anti-oxidative and anti-inflammatory properties [14]. The most important advantages of Triphala include easy access, low cost, long-term substantivity, less toxicity and absence of microbial resistance [16]. The present experimental study was designed to evaluate and compare the antibacterial activity of Triphala and 0.5, and 1, 2.5 and 5% concentrations of NaOCl against *E. faecalis*.

## Methods and Materials

Two hundred plates containing agar medium, Mueller-Hinton broth (MHB) (Difco Laboratories, Detroit, MI, USA) and 5% sterile sheep blood were prepared. To confirm the sterility of the culture media, the plates were incubated for 24 h at 37^°^C under aerobic conditions and were then divided into 5 experimental groups (*n*=38) and two positive and negative control groups; group 1; Triphala, group 2; 0.5% NaOCl, group 3; 1% NaOCl, group4; 2.5 % NaOCl and group 5; 5% NaOCl.

Triphala powder (IMPCOPS Ltd, Chennai, India) was dissolved in 10% dimethylsulfoxid (SD Fine Chemicals, Chennai, India) to prepare an irrigation solution at a concentration of 5 mg/ml. Different concentrations of NaOCl were prepared by dilution of 5.25% solution with sterile water without preservatives.

A 0.5 McFarland suspension of the reference *E. faecalis* bacterium (ATCC 299212) was prepared in the brain-heart infusion (BHI) broth (Difco Laboratories, Detroit, MI, USA) culture media and inoculated onto the prepared culture media. The culture was then kept at -20°C in a freezer. Single-well plates with the diameter of 6 mm and depth of 4 mm were prepared and 10 mL of the each test solution was pipetted into each well. The cultured *E. faecalis* was then defrozen and carried on the solid BHI agar enriched with 7% sterile sheep blood and incubated at 37^°^C and 5% CO_2_ gas for 24 h. The formed colonies were again transferred into MHB and incubated at 37^°^C under aerobic conditions for 24 h. Subsequently, spectrophotometry was used to prepare a standard suspension of *E. faecalis* in the MHB containing 1.5×10^8^ (CFU/mL) bacteria in each mm equivalent to 0.5 McFarland standard. The suspension was homogeneously spared onto the surface of MHB agar containing sheep blood by using a sterile cotton swab. Positive and negative controls were also prepared, maintaining the plates inoculated and without inoculum and sterile saline as irrigant solution, for similar time intervals and under identical incubation conditions. All assays were carried out under aseptic conditions. The plates were incubated for 1 week at 37^°^C in a moist environment with 5% CO_2_ gas. Finally, the diameter of the halo formed around each disk (zone of inhibition) was measured using a glass ruler in mm by calculating the shortest distance between the outer margin of the material and the initial point of bacterial growth. The Kruskal-Wallis test was used to compare the mean diameter of the inhibition zones among the tested materials and the Mann-Whitney U test was used for the two-by-two comparison of the groups. The level of significance was set at 0.05.

## Results

The mean diameters of microbial inhibition zone was 7.3±1.3, 4.6±1.6, 6.3±1.2, 7±1 and 7.6±1.1 mm in the Triphala and 0.5, 1, 2.5 and 5% NaOCl groups, respectively. All positive samples displayed microbial growth and all negative samples yielded negative cultures. The Kruskal-Wallis test showed significant differences between the groups (*P*=0.002). The Mann-Whitney U test also showed significant differences in antibacterial activity of Triphala compared with 0.5% (*P*=0.003) and 1% NaOCl solution (*P*=0.001). However, there were no significant differences in the antimicrobial properties of Triphala and 2.5% (*P*=0.20) and 5% NaOCl solutions (*P*=0.28).

## Discussion

This experimental study compared the antibacterial properties of a herbal endodontic irrigation (Triphala) with different concentrations of NaOCl on *E. faecalis *and showed that the antimicrobial properties of Triphala and 2.5 and 5% NaOCl were comparable.

The principal aim of endodontic treatment is to prevent or eliminate microbial contamination of the root canal system [1, 2], and the main reason for the majority of treatment failures is persistence of infections within these spaces. Although mechanical instrumentation and use of irrigation solutions with strong antimicrobial properties eliminate the majority of intracanal microorganisms, it has been demonstrated that it is not possible to completely eliminate microorganisms [6, 7, 10]. On the other hand, some microorganisms are resistant to antimicrobial agents used within the root canal [4]. *E. faecalis* is a gram-positive facultative anaerobic microorganism, which has been isolated from almost 38% of teeth with failed endodontic treatment [17]. It is resistant to CH which is the most commonly used intracanal antimicrobial agent [4, 10]. This microorganism has been used in a large number of studies for the evaluation of antimicrobial properties due to its role in retreatment failure. *E. faecalis *can survive even in obturated canals without support from other microorganisms or with very small amounts of nutrients [17].

NaOCl is currently the most commonly used intracanal irrigation solution at various concentrations [9, 11]. It has a broad antimicrobial activity against endodontic microorganisms and biofilms, including difficult-to-eliminate species like *Enterococci*, *Acitinomycetes* and *Candida albicans* [5, 18]. It is demonstrated that 2.5% NaOCl can reduce the intracanal bacteria by 90% [19]. In another study 5.25% NaOCl displayed the most efficient antibacterial action and had significantly greater substantivity at different time intervals [20]. Studies evaluating cytotoxicity of NaOCl have shown higher cytotoxicity and caustic effects of 5.25% NaOCl compared to its 0.5 and 1% concentrations on healthy tissues [9, 11]. In many countries concerns about the chemical and toxic effects of the solution have resulted in the use of 0.5 and 1% concentrations of NaOCl as an intracanal irrigation solution instead of 5.25% concentration [6, 8, 21].

In recent years, there has been an increased tendency to use plant-derived alternative irrigation solutions with pharmaceutical properties. Previous studies regarding the comparison of antimicrobial activities of Triphala and NaOCl have used 3 and 5% concentrations of NaOCl [11, 14]. In the present study low concentrations of 0.5 and 1% NaOCl were used because they are safe and studies have shown no significant differences in the antimicrobial activity between 1 and 5% NaOCl solutions [11, 22].

The results of the present study showed higher antimicrobial activity of Triphala compared to 0.5 and 1% NaOCl and that it can be used as an appropriate irrigation solution in endodontics given the advantages of natural medications and the disadvantages of NaOCl. This claim needs more investigation. Another difference between this study and previous studies is the fact that previous studies have not used the biofilms of* E. faecalis*. Biofilms are more resistant to antibacterial agents compared to planktonic bacteria [17]. Therefore, conducting similar studies with biofilms* of E. faecalis* is suggested in order to compare the antimicrobial activity of Triphala with lower concentrations of NaOCl solution.

## Conclusion

Under the circumstances of this *in vitro *study, Triphala was more effective on cultures of *E. faecalis *compared to 0.5 and 1% NaOCl.
